# Peripheral Blood Lymphocyte Subsets Predict the Efficacy of Immune Checkpoint Inhibitors in Non–Small Cell Lung Cancer

**DOI:** 10.3389/fimmu.2022.912180

**Published:** 2022-07-01

**Authors:** Kang Miao, Xiaotong Zhang, Hanping Wang, Xiaoyan Si, Jun Ni, Wei Zhong, Jing Zhao, Yan Xu, Minjiang Chen, Ruili Pan, Mengzhao Wang, Li Zhang

**Affiliations:** Department of Pulmonary and Critical Care Medicine, Peking Union Medical College Hospital, Chinese Academy of Medical Sciences & Peking Union Medical College, Beijing, China

**Keywords:** immune checkpoint inhibitors (ICIs), lymphocyte subsets, CD4^+^CD45RA^−^ T cell, biomarker, CD8^+^CD38^+^ T cell

## Abstract

**Background:**

Non–small cell lung cancer (NSCLC) has entered the era of immunotherapy. However, only partial patients were able to benefit from immune checkpoint inhibitors (ICIs). Currently, biomarkers for predicting patients’ response to ICIs are primarily tumor tissue dependent and have limited accuracy. There is an urgent need to explore peripheral blood-based biomarkers to predict the efficacy and safety of ICI therapy.

**Methods:**

To explore the correlation between lymphocyte subsets and the efficacy and safety of ICIs, we retrospectively analyzed peripheral blood lymphocyte subsets and survival prognosis data of 136 patients with stage IV NSCLC treated with ICIs.

**Results:**

The two factors that had the greatest impact on the prognosis of patients with NSCLC treated with ICIs were CD4^+^CD45RA^−^ T cell (HR = 0.644, P = 0.047) and CD8^+^ T/lymphocyte (%) (HR = 1.806, P = 0.015). CD4^+^CD45RA^−^ T cell showed excellent predictive efficacy (AUC = 0.854) for ICIs monotherapy, with a sensitivity of 75.0% and specificity of 91.7% using CD4^+^CD45RA^−^ T cell >311.3 × 10^6^/L as the threshold. In contrast, CD8^+^ T/lymphocyte (%) was only associated with the prognosis but had no predictive role for ICI efficacy. CD4^+^ T cell and its subsets were significantly higher in patients with mild (grades 1–2) immune-related adverse events (irAEs) than those without irAEs. CD8^+^CD38^+^ T cell was associated with total irAEs and severe (grades 3–4) irAEs but was not suitable to be a predictive biomarker.

**Conclusion:**

Peripheral blood CD4^+^CD45RA^−^ T cell was associated with the prognosis of patients with NSCLC applying ICIs, whereas CD8^+^CD38^+^ T cell was associated with irAEs and severe irAEs.

## Background

Immune checkpoint inhibitors (ICIs), represented by cytotoxic T lymphocyte-associated antigen 4 (CTLA-4) inhibitors, programmed cell death protein 1 (PD-1) inhibitors, and programmed cell death protein ligand 1 (PD-L1) inhibitors, have transformed cancer treatment since ipilimumab was first approved for melanoma in 2011 (1). ICIs bring lasting objective remission to patients with cancer by activating autologous lymphocytes. Currently, they have been approved for multiple indications including melanoma, non–small cell lung cancer (NSCLC), and head and neck squamous carcinoma. The approved ICIs in NSCLC are mainly PD-1 inhibitors, including pembrolizumab, nivolumab, camrelizumab, and tislelizumab (2). Although PD-L1 is currently the best biomarker for predicting the efficacy of ICIs in patients with NSCLC, the accuracy is still limited. However, not all patients with NSCLC can benefit from ICI therapy. Keynote-042 study showed that only 27.3% patients with positive PD-L1 were able to achieve an objective response from pembrolizumab monotherapy. In addition, the objective response rate (ORR) was only 39.1% even in those with PD-L1 ≥ 50% (3). Nevertheless, patients with low or negative PD-L1 expression may also achieve favorable efficacy from immunotherapy (4). In addition, current biomarkers for predicting patients’ response to ICIs are primarily tumor tissue dependent. Therefore, exploring new biomarkers that can predict the efficacy and safety of ICIs has become an urgent need.

The key factor for immunotherapy is to activate the lymphocytes; thus, the quantity and subset of lymphocytes are closely related to the efficacy of ICIs. T cell constitute the main effector cells involved in the immune response, which can be generally classified into cytotoxic T cell (CD8^+^) and regulatory T cell (CD4^+^). By detecting CD45RA, CD28, and CD38 molecules on the surface of T cell, they can be further categorized into memory subsets, functional subsets, and activated subsets (5). CD8^+^ T cells are essential participants in the anti-tumor immune response for their cytotoxicity and ability to migrate from peripheral blood into tumor tissues (6). Studies have shown that the counts of infiltrating CD8^+^ T cell were intimately associated with the antitumor efficacy of PD-1 inhibitors (7). In contrast to the direct tumor-killing effect of CD8^+^ T cells, CD4^+^ T cells play more of an immune-modulatory and paracrine role. CD4^+^ Th1 cells promote the differentiation of initial CD8^+^ T cell into cytotoxic T lymphocytes (CTL) through the CD70-CD27 pathway and secreting cytokines, such as interferon γ (IFN-γ) and interleukin 2 (IL-2) (8). Recently, several clinical studies have shown that the peripheral blood CD4^+^ T subsets may be associated with tumor objective response for immunotherapy (9, 10).

Herein, we conducted a retrospective study to analyze the correlation between baseline peripheral blood lymphocyte subsets and tumor regression after ICI therapy to explore the applicability of lymphocyte subsets to be a potential biomarker.

## Methods

### Study Objectives and Ethics Approval Statement

Our retrospective study collected baseline lymphocyte subset data and the follow-up information of 136 patients with stage IV NSCLC treated with ICIs. The aim was to explore the correlation between lymphocyte subsets and the therapeutic effect of ICIs and to find a new biomarker for ICI therapy. The study design was in accordance with the ethical guidelines of the Declaration of Helsinki, and ethical approval was obtained from the Ethical Review Committee of Peking Union Medical College Hospital. Informed consent was waived due to the retrospective character.

### Inclusion and Exclusion Criteria

Inclusion criteria: (i) patients were pathologically confirmed NSCLC; (ii) the disease stage was stage IV; (iii) patients utilized ICIs during the course of treatment; and (iv) lymphocyte subsets were detected within 28 days before the first dose of ICIs. Exclusion criteria: (i) the follow-up period after ICI therapy <6 months; and (ii) received two or more types of ICI therapy.

### Data Collection

For each patient who met the inclusion criteria, we obtained the following information separately: (i) baseline lymphocyte subset data, including total lymphocytes, B cell, T cell, NK cells, CD4^+^ T cell, CD8^+^ T cell, CD4^+^CD45RA^−^ T cell, CD4^+^CD45RA^+^ T cell, CD4^+^CD28^+^ T cell, CD8^+^CD28^+^ T cell, CD8^+^DR^+^ T cell, CD8^+^CD38^+^ T cell, CD4^+^ T cell/lymphocyte (%), and CD8^+^ T cell/lymphocyte (%); (ii) patient basic information, including gender, age, tumor pathology, history of smoking, history of alcohol consumption, and eastern Cooperative Oncology Group performance status (ECOG PS) score; (iii) treatment details, including the lines of ICIs applied and whether combined with chemotherapy; (iv) efficacy and safety assessment, including ORR, progression-free survival (PFS), and irAEs.

### Data Analysis

IBM SPSS version 24.0 was used for statistical analysis, and GraphPad Prism version 8.0.1 was used to graph the statistics. Median and interquartile range (IQR) were used for description of baseline lymphocyte subsets due to non-conformance with normal distribution. Patients with disease control and PFS ≥6 months were recorded as benefit group, whereas those with progressive disease (PD) or PFS <6 months were recorded as non-benefit group. Comparisons of the average values were analyzed using the Mann–Whitney test. Patient basic information and treatment details were described as count values and percentages. The receiver operating characteristic (ROC) curve was established to assess the value of efficacy prediction. CD4^+^CD45RA^−^ T cell and CD8^+^ T/lymphocyte (%) were divided into high-level group and low-level group according to the median. Correlations between them and patient basic information were analyzed by the chi-square test. Kaplan–Meier survival curves were used to reflect differences in survival benefit. Log-rank test was used to explore the effect of basic information, treatment details, and baseline lymphocyte subsets on the efficacy of ICIs. Logistic regression was used to analyze the risk factors for irAEs and severe irAEs. All independent variables were included in the univariate regression, and then indicators that reached the threshold (P < 0.2) were included into multivariate regression. P < 0.05 was determined to be with statistical difference.

## Results

### Basic Characteristics of the Patients

A total of 136 patients with stage IV NSCLC met the inclusion and exclusion criteria. Among them, 94 were male and 42 were female, with a median age of 64 years old. There were more patients with non-squamous NSCLC (75 patients) than squamous NSCLC (61 patients). The majority of patients (71 patients) chose ICIs for first-line treatment, whereas 47 patients (34.6%) for the second line and 10 patients (13.2%) for the third line and beyond. Only a quarter of patients chose ICIs as monotherapy (de-chemotherapy), which means that most of patients applied ICIs combined with chemotherapy. There were 61.8% patients had smoking history and 29.4% had alcohol consumption history. Most of patients were in good physical condition on ICIs, 90.5% patients were of 0-1 ECOG PS scores **(** **Table 1** **)**. The overall ORR of the patients was 42.6%, with a median PFS of 8.5 months. Immune-related adverse events (irAEs) occurred in 49 (36.0%) patients, of which 24 (17.6%) were grade 3 or higher.

**Table 1:** Basic information.

Basic Information	Number
**Patients**	136
**Sex**	
male	94 (69.1%)
female	42 (30.9%)
**Age**	64 (60–70)
<60	33 (24.3%)
≥60	103 (75.7%)
**Histology**	
Non-squamous carcinoma	75 (55.1%)
Squamous carcinoma	61 (44.9%)
**Line of therapy**	
First line	71 (52.2%)
Second line	47 (34.6%)
Third line and beyond	10 (13.2%)
**Combined chemotherapy**	
No	32 (23.5%)
Yes	104 (76.5%)
**Smoking status**	
No	52 (38.2%)
Yes	84 (61.8%)
**Drinking status**	
no	96 (70.6%)
yes	40 (29.4%)
**ECOG PS**	
0	70 (51.5%)
1	53 (39.0%)
2–4	13 (9.6%)
**Total lymphocyte (×10^6^/L)**	1295.2 (973.1–1728.4)
**B cell (×10^6^/L)**	94.7 (57.1–175.6)
**T cell (×10^6^/L)**	916.6 (639.4–1221.1)
**NK cell (×10^6^/L)**	258 (164.4–360.1)
**CD4^+^ T (×10^6^/L)**	475.9 (302.4 - 668)
**CD8^+^ T (×10^6^/L)**	340.1 (237.5 - 493.3)
**CD4^+^CD45RA^−^ T (×10^6^/L)**	348.3 (218.3 - 510.1)
**CD4^+^CD45RA^+^ T (×10^6^/L)**	109.1 (49.4 - 189.1)
**CD4^+^CD28^+^ T (×10^6^/L)**	413.1 (257.7 - 613.7)
**CD8^+^CD28^+^ T (×10^6^/L)**	152.2 (94.9 - 219.7)
**CD8^+^DR^+^ T (×10^6^/L)**	176.3 (112.3 - 264.2)
**CD8^+^CD38^+^ T (×10^6^/L)**	151.7 (91.3 - 208.9)
**CD4^+^ T/lymphocyte (%)**	36.1 (29.1 - 45.4)
**CD8^+^ T/lymphocyte (%)**	26.9 (21.7 - 35.4)

### Correlation of Lymphocyte Subsets With the Efficacy of ICIs

To explore the relationship between baseline lymphocyte subsets and the efficacy of ICIs, patients were divided into two groups based on survival benefit. Patients with disease control and PFS ≥6 months were recorded as benefit group, whereas those with PD or PFS <6 months were recorded as non-benefit group. The counts of total lymphocytes and all lymphocyte subsets were higher in the benefit group than in the non-benefit group. Among them, total lymphocytes, B cell, T cell, CD4^+^ T cell, various CD4^+^ T cell subsets (CD4^+^CD45RA^−^ T cell, CD4^+^CD45RA^+^ T cell, and CD4^+^CD28^+^ T cell), and CD8^+^CD28^+^ T cell showed statistical differences (P < 0.05) **(** **Figure S1** **)**. In addition, we explored changes in lymphocyte subsets after two cycles of ICI treatment. However, only total T cells showed a decreasing trend in the benefit group **(** **Figure S2** **)**. Patient basic information was included into univariate cox regression analysis together with baseline lymphocyte subsets **(** **Table 2** **)**. The results showed that gender (P = 0.196), age (P = 0.191), type of pathology (P = 0.080), lines of ICIs usage (P = 0.199), ECOG PS score (P = 0.064), B cell (P = 0.174), CD4^+^CD45RA^−^ T cell (P = 0.016), and CD8^+^ T/lymphocyte (%) (P = 0.006) may contribute to the prognosis (threshold: P < 0.2). Multivariate cox regression showed that the two factors with the greatest impact were CD4^+^CD45RA^−^ T cell (HR = 0.644, P = 0.047) and CD8^+^ T/lymphocyte (%) (HR = 1.806, P = 0.015), respectively **(** **Table 3** **)**.

**Table 2:** Univariate Cox regression of PFS.

		HR	95% CI	P-value
**Sex**	Male/female	1.337	0.861-2.075	0.196
**Age**	<60/≥60	1.412	0.842-2.37	0.191
**Histology**	Non-squamous/squamous	0.686	0.45-1.046	0.080
**Line of therapy**	First/second/third and beyond	1.220	0.883-1.686	0.199
**Combined chemotherapy**	No/yes	1.155	0.696-1.919	0.577
**Smoking status**	No/yes	0.782	0.513-1.193	0.254
**Drinking status**	No/yes	1.062	0.683-1.652	0.789
**ECOG PS**	0/1/2-4	1.338	0.984-1.819	0.064
**Total lymphocyte**	×10^6^	0.788	0.519-1.197	0.264
**B cell**	×10^6^	0.749	0.493-1.136	0.174
**T cell**	×10^6^	0.972	0.641-1.475	0.894
**NK cell**	×10^6^	0.781	0.517-1.178	0.238
**CD4^+^ T**	×10^6^	0.785	0.517-1.191	0.255
**CD8^+^ T**	×10^6^	1.150	0.758-1.745	0.511
**CD4^+^CD45RA^−^ T**	×10^6^	0.599	0.395-0.907	0.016
**CD4^+^CD45RA^+^ T**	×10^6^	1.016	0.67-1.54	0.941
**CD4^+^CD28^+^ T**	×10^6^	0.803	0.53-1.216	0.300
**CD8^+^CD28^+^ T**	×10^6^	0.842	0.556-1.274	0.416
**CD8^+^DR^+^ T**	×10^6^	1.021	0.673-1.549	0.922
**CD8^+^CD38^+^ T**	×10^6^	1.049	0.691-1.591	0.823
**CD4^+^ T/lymphocyte**	×100%	0.825	0.548-1.248	0.363
**CD8^+^ T/lymphocyte**	×100%	1.803	1.182-2.750	0.006

**Table 3:** Multivariate Cox regression of PFS.

		Median PFS	HR	95% CI	P-value
**Sex**	Male	8.6 months	Reference		
	Female	7.5 months	1.203	0.718–2.015	0.483
**Age**	<60	11.8 months	Reference		
	≥60	8.1 months	1.252	0.726–2.161	0.419
**Histology**	Non-squamous	7.5 months	Reference		
	Squamous	11.7 months	0.793	0.483–1.303	0.360
**Line of therapy**	First line	10.5 months	Reference		
	Second line	7 months	0.938	0.558–1.576	0.810
	Third line and beyond	5.9 months	1.380	0.603–3.157	0.446
**ECOG PS**	0	11.5 months	Reference		
	1	7.5 months	1.219	0.775–1.918	0.392
	2–4	5.5 months	1.131	0.510–2.508	0.762
**B cell**	Low	6.2 months	Reference		
	High	11.2 months	1.035	0.642–1.668	0.887
**CD4^+^CD45RA^−^ T**	Low	5.9 months	Reference		
	High	11.8 months	0.644	0.417–0.994	0.047
**CD8^+^ T/lymphocyte (%)**	Low	11.8 months	Reference		
	High	6.3 months	1.806	1.122–2.905	0.015

**Figure 1:**
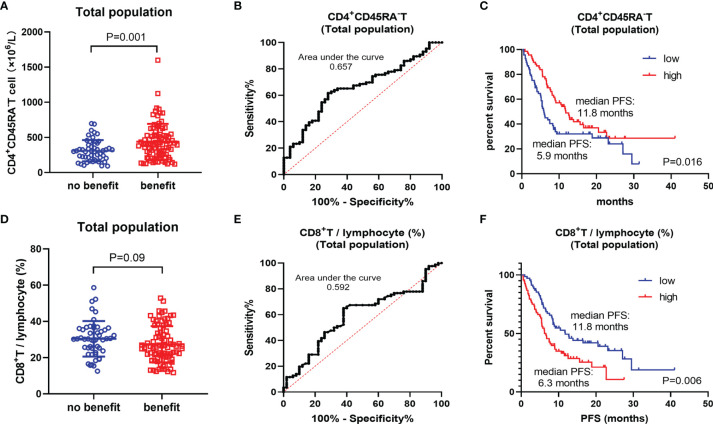
Total population analysis. **(A)** Differences of CD4^+^CD45RA^−^ T cell between benefit group and non-benefit group. **(B)** ROC curve of CD4^+^CD45RA^−^ T cell. **(C)** Kaplan–Meier survival curve of different levels of CD4^+^CD45RA^−^ T cell. **(D)** Differences of CD8^+^ T/lymphocyte (%) between benefit group and non-benefit group. **(E)** ROC curve of CD8^+^ T/lymphocyte (%). **(F)** Kaplan–Meier survival curve of different levels of CD8^+^ T/lymphocyte (%).

Further, we performed a systematic analysis for these two biomarkers. Baseline CD4^+^CD45RA^−^ T cells (P = 0.001) were significantly higher in the benefit group, whereas CD8^+^ T/lymphocytes (%) were lower in the benefit group (P = 0.09) **(** **Figures 1A, D** **)**. However, they were not ideal biomarker for predicting the prognosis of patients with NSCLC, with the area under the curve (AUC) of the ROC being just 0.657 and 0.592 **(** **Figures 1B, E** **)**. Kaplan–Meier survival curve showed that the high CD4^+^CD45RA^−^ T-cell group (median PFS: 11.8 months) had a significantly better PFS benefit than the low CD4^+^CD45RA^−^ T-cell group (median PFS: 5.9 months) (P = 0.016). There was also a difference in survival prognosis between the high CD8^+^ T/lymphocyte (%) group (median PFS: 6.3 months) and the low CD8^+^ T/lymphocyte (%) group (median PFS: 11.8 months) (P = 0.006) but showing an opposite trend to CD4^+^CD45RA^−^ T cell **(** **Figures 1C, F** **)**.

### Subgroup Analysis

**Figure 2:**
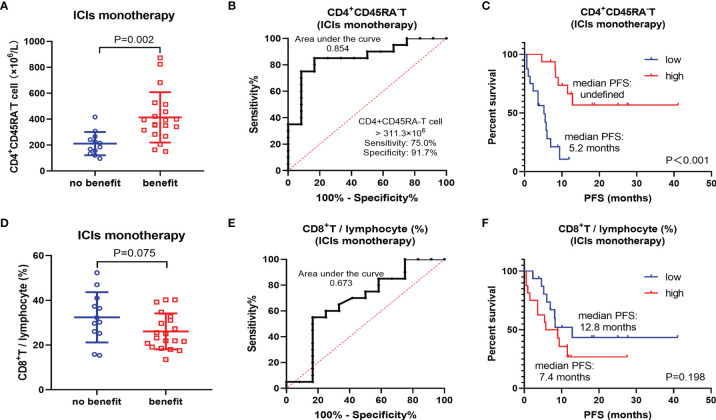
Subgroup analysis of ICIs monotherapy. **(A)** Differences of CD4^+^CD45RA^−^ T cell between benefit group and non-benefit group. **(B)** ROC curve of CD4^+^CD45RA^−^ T cell. **(C)** Kaplan–Meier survival curve of different levels of CD4^+^CD45RA^−^ T cell. **(D)** Differences of CD8^+^ T/lymphocyte (%) between benefit group and non-benefit group. **(E)** ROC curve of CD8^+^ T/lymphocyte (%). **(F)** Kaplan–Meier survival curve of different levels of CD8^+^ T/lymphocyte (%).

Considering that the patients included in this study were treated with ICIs monotherapy and ICIs combined with chemotherapy. The analysis of total population only represents the survival prognosis, but not the efficacy of ICIs. Therefore, a subgroup of 32 patients treated with ICIs monotherapy was selected for further analysis. In this subgroup, CD4^+^CD45RA^−^ T cell showed a more significant difference (p = 0.002) between the benefit and non-benefit groups **(** **Figure 2A** **)**. Notably, its efficacy as a biomarker to predict the therapeutic benefit of ICIs is also excellent (AUC = 0.854), with a sensitivity of 75.0% and specificity of 91.7% using CD4^+^CD45RA^−^ T cell >311.3 × 10^6^/L as the threshold **(** **Figure 2B** **)**. The results of Kaplan–Meier survival curves also demonstrated close relationship between CD4^+^CD45RA^−^ T cell and the efficacy of ICIs. The median PFS was not reached in the CD4^+^CD45RA^−^ T-cell high group and 5.2 months in the CD4^+^CD45RA^−^ T-cell low group (P < 0.001) **(** **Figure 2C** **)**. However, CD8^+^ T/lymphocyte (%) did not show a better correlation with the efficacy of ICIs, with no significant difference (P = 0.075) **(** **Figure 2D** **)**. In addition, the AUC was only 0.673 of CD8^+^ T/lymphocyte (%), which was considerably lower than that of CD4+CD45RA^−^ T cell **(** **Figure 2E** **)**. There was also no meaningful discrepancy observed between the high-level group and the low-level group of CD8^+^ T/lymphocyte (%) on the Kaplan–Meier survival curve (P = 0.198) **(** **Figure 2F** **)**.

Further, we analyzed CD4^+^CD45RA^−^ T cell and CD8^+^ T/lymphocyte (%) with patient basic information, separately. The results showed that CD8^+^ T/lymphocytes (%) were well matched for all basic information, whereas CD4^+^CD45RA^−^ T cells were matched for gender, age, pathology type, combination of chemotherapy, smoking history, alcohol consumption history, and ECOG PS score but had strong relevance with lines of ICIs usage (p = 0.003) **(** **Table 4** **)**. The count of CD4^+^CD45RA^−^ T cell was higher in the first-line treatment than in the second line and beyond (P < 0.001) **(** **Figure S3** **)**.

**Table 4:** Basic information matching analysis.

	CD4^+^CD45RA^−^ T cell		P-value	CD8^+^ T/lymphocyte (%)		P-value
	Low	High		Low	High	
**Patients**	68	68		68	68	
**Sex**			1.000			0.710
Male	47 (69.1%)	47 (69.1%)		46 (67.6%)	48 (70.6%)	
Female	21 (30.9%)	21 (30.9%)		22 (32.4%)	20 (29.4%)	
**Age**			0.424			0.161
<60	14 (20.6%)	19 (27.9%)		20 (29.4%)	13 (19.1%)	
≥60	54 (79.4%)	49 (72.1%)		48 (70.6%)	55 (80.9%)	
**Histology**			0.168			0.605
Non-squamous carcinoma	42 (61.8%)	33 (48.5%)		39 (57.4%)	36 (52.9%)	
Squamous carcinoma	26 (38.2%)	35 (51.5%)		29 (42.6%)	32 (47.1%)	
**Line of therapy**			0.003			0.765
First line	30 (44.1%)	49 (72.1%)		38 (55.9%)	41 (60.3%)	
Second line	30 (44.1%)	17 (25.0%)		24 (35.3%)	23 (33.8%)	
Third line and beyond	8 (11.8%)	2 (2.9%)		6 (8.8%)	4 (5.9%)	
**Combined chemotherapy**			0.419			1.000
No	18 (26.5%)	14 (20.6%)		16 (23.5%)	16 (23.5%)	
Yes	50 (73.5%)	54 (79.4%)		52 (76.5%)	52 (76.5%)	
**Smoking status**			0.217			0.158
No	30 (44.1%)	22 (32.4%)		30 (44.1%)	22 (32.4%)	
Yes	38 (55.9%)	46 (67.6%)		38 (55.9%)	46 (67.6%)	
**Drinking status**			0.572			0.707
No	50 (73.5%)	46 (67.6%)		49 (72.1%)	47 (69.1%)	
Yes	18 (26.5%)	22 (32.4%)		19 (27.9%)	21 (30.9%)	
**ECOG PS**			0.625			0.293
0	33 (48.5%)	37 (54.5%)		38 (55.9%)	32 (47.1%)	
1	27 (39.7%)	26 (38.2%)		26 (38.2%)	27 (39.7%)	
2–4	8 (11.8%)	5 (7.4%)		4 (5.9%)	9 (13.2%)	

**Figure 3:**
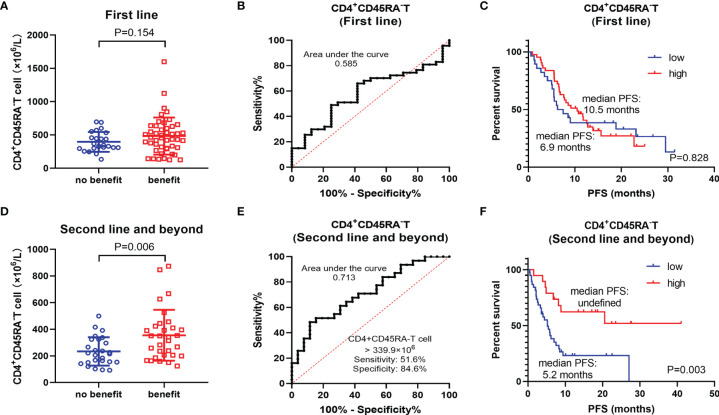
Subgroup analysis of different treatment lines. **(A)** Differences of CD4^+^CD45RA^−^ T cell between benefit group and non-benefit group in the first-line patients. **(B)** ROC curve of CD4^+^CD45RA^−^ T cell in the first-line patients. **(C)** Kaplan–Meier survival curve of different levels of CD4^+^CD45RA^−^ T cell in the first-line patients. **(D)** Differences of CD4^+^CD45RA^−^ T cell between the benefit group and the non-benefit group in the second-line and beyond patients. **(E)** ROC curve of CD4^+^CD45RA^−^ T cell in the second-line and beyond patients. **(F)** Kaplan–Meier survival curve of different levels of CD4^+^CD45RA^−^ T cell in the second-line and beyond patients.

In the population of first-line treatment, there was no remarkable difference in CD4^+^CD45RA^−^ T cell between the benefit and non-benefit groups (P = 0.154) **(** **Figure 3A** **)**. ROC curves showed that CD4^+^CD45RA^−^ T-cell count was not an ideal biomarker (AUC = 0.585) **(** **Figure 3B** **)**. Kaplan–Meier survival curves also indicated no significant prognostic difference between the high CD4^+^CD45RA^−^ T-cell group and the low CD4^+^CD45RA^−^ T-cell group (P = 0.828) **(** **Figure 3C** **)**. These findings differ dramatically from those of total population. Therefore, we considered whether the survival benefit in the high CD4^+^CD45RA^−^ T-cell group was more likely to originate from the second-line and beyond population. In this subgroup, CD4^+^CD45RA^−^ T-cell count was significantly higher in the benefit group than in the non-benefit group (P = 0.006) **(** **Figure 3D** **)**. The AUC was 0.713, at the threshold of CD4^+^CD45RA^−^ T cell >339.9 × 106/L, the sensitivity was 51.6%, and the specificity was 84.6% **(** **Figure 3E** **)**. The high CD4^+^CD45RA^−^ T-cell group exhibited a notable PFS benefit (median PFS: undefined) compared with the low CD4^+^CD45RA^−^ T-cell group (median PFS: 5.2 months), P = 0.003 **(** **Figure 3F** **)**.

### Correlation of Lymphocyte Subsets With irAEs

In addition, we explored the correlation between peripheral blood lymphocyte subsets and all kinds of irAEs **(** **Figure S4** **)**. We found that baseline counts of total lymphocytes, T cell, CD4^+^ T cell, and various CD4^+^ T-cell subsets (CD4^+^CD45RA^−^ T cell, CD4^+^CD45RA^+^ T cell, and CD4^+^CD28^+^ T cell) were higher in the mild (grades 1–2) irAEs group than in the non-irAEs group. However, the expression of these lymphocyte subsets in the severe (grades 3–4) irAEs group remained at a low level, similar to that in the non-irAEs group. CD8^+^CD38^+^ T cells were the only cell subset that showed statistical differences in the populations of severe irAEs and non-irAEs. Logistic regression analysis showed that the total T cells and CD8^+^CD38^+^ T cells were strongly associated with the development of irAEs, whereas CD4^+^ T-cell subsets did not show statistical contributions to irAEs. In addition, CD8^+^CD38^+^ T cells were also associated with the development of severe irAEs, P = 0.05 (**Table 5**). However, the ROC curve constructed with CD8^+^CD38^+^ T predicting the occurrence of severe irAEs had an AUC of only 0.535, which was not suitable to be a predictive biomarker **(** **Figure S5** **)**.

**Table 5:** Logistic regression of irAEs.

		irAEs (P-value)		Severe irAEs (P-value)
		Univariate	Multivariate	Univariate
**Sex**	Male/female	0.110	0.579	0.492
**Age**	<60/≥60	0.229		0.138
**Histology**	Non-squamous/squamous	0.765		0.188
**Line of therapy**	First/second/third and beyond	0.191	0.412	0.779
**Combined chemotherapy**	No/yes	0.603		0.500
**Smoking status**	No/yes	0.170	0.750	0.586
**Drinking status**	No/yes	0.534		0.977
**ECOG PS**	0/1/2–4	0.453		0.529
**Total lymphocyte**	×10^6^	0.090	0.229	0.536
**B cell**	×10^6^	0.332		0.925
**T cell**	×10^6^	0.120	0.017	0.502
**NK cell**	×10^6^	0.410		0.870
**CD4^+^ T**	×10^6^	0.050	0.123	0.552
**CD8^+^ T**	×10^6^	0.777		0.607
**CD4^+^CD45RA^−^ T**	×10^6^	0.213		0.415
**CD4^+^CD45RA^+^ T**	×10^6^	0.009	0.206	0.911
**CD4^+^CD28^+^ T**	×10^6^	0.101	0.599	0.747
**CD8^+^CD28^+^ T**	×10^6^	0.678		0.540
**CD8^+^DR^+^ T**	×10^6^	0.538		0.699
**CD8^+^CD38^+^ T**	×10^6^	0.036	0.011	0.050
**CD4^+^ T/lymphocyte**	×100%	0.905		0.491
**CD8^+^ T/lymphocyte**	×100%	0.219		0.924

## Discussion

Currently, biomarkers for predicting the efficacy of ICIs mainly include PD-L1, tumor mutational burden (TMB), microsatellite instability (MSI), and gene expression profile of tumor-infiltrating lymphocytes (TILs) (11). In the field of NSCLC, PD-L1 is currently the only biomarker approved by the Food and Drug Administration (FDA) (12). However, its credibility remains to be controversial. In addition, mainstream biomarkers are based on the acquisition of tumor tissues. No relevant peripheral blood biomarkers have been approved by the FDA, European Medicines Agency (EMA), and Pharmaceuticals and Medical Devices Agency (PMDA) (13, 14). However, the accessibility of tissue is limited, especially for patients of second line and beyond. Therefore, peripheral blood lymphocyte may be a promising non-invasive biomarker for patients treated with ICIs.

In this study, we explored the predictive effect of peripheral blood lymphocyte subsets on the efficacy of ICIs. We found that CD4^+^CD45RA^−^ T cells and CD8^+^ T/lymphocytes (%) were associated with the prognosis of patients with NSCLC treated with ICIs. Low CD4^+^CD45RA^−^ T cell was associated with poor prognosis, whereas low CD8^+^ T/lymphocyte (%) represented better prognosis. In the further subgroup analysis of the ICIs monotherapy population, we observed that only CD4^+^CD45RA^−^ T cell reflected the efficacy of ICIs, whereas CD8^+^ T/lymphocyte (%) merely correlated with the prognosis. In addition, we explored the correlation between lymphocyte subsets and irAEs. The results showed that CD4^+^ T-cell subsets were higher in the mild-irAEs group than in the non-irAEs group but did not contribute significantly in logistic regression. CD8^+^CD38^+^ T cell was associated with irAEs and severe irAEs but was not suitable as a predictive marker.

After activation by specific antigens, T cell would form two subtypes that differ in function, called effector T cell and memory T cell. Memory T cell, after secondary activated by antigen, would infiltrate into tumor tissue and express perforin to induce tumor cells apoptosis (15). CD45RA, one of the surface features of initial T cell, is expressed by initial T cell in the thymus and would disappear after antigen stimulation (16). It means that CD45RA^−^ is the characteristic of memory T cell. CD62L, the characteristic of peripheral memory T cell, is expressed by central memory T cell but peripheral memory T cell (17). Kagamu et al. concluded that CD4^+^CD62L^−^ T cell presented in higher baseline counts in the responders to ICIs. In addition, patients who maintained high levels of CD62L^−^CD4^+^ T cell had a prolonged survival, compared with those patients whose CD62L^−^CD4^+^ T-cell counts decreased after ICI therapy (18). Similar conclusion was obtained by Zuazo et al. that patients with large amounts of highly differentiated (CD27^−^CD28^−^) CD4^+^ T cell responded well to ICI therapy. ROC curves constructed with it were of good predictive value for the efficacy of ICIs (AUC = 0.85) (19). The above two studies demonstrated from two perspectives of peripheral memory T cell and highly differentiated T cell, respectively. We reaffirmed this idea from the third perspective that baseline memory CD4^+^ T cell (central and peripheral) can reflect the efficacy of ICIs. In the subgroup of ICIs monotherapy patients, the ROC curve of CD45RA^−^CD4^+^ T cell had an AUC of 0.854, which was in favorable consistency with the study of Zuazo et al.

With the widespread application of ICIs in multiple tumor types, the indications approved have been gradually pushed to the first line from the second line and beyond (20). However, it remains to be unclear whether the application of ICIs in the first line brings better overall survival (OS) benefit. Chemotherapy and other anti-neoplastic therapy would cause myelosuppression, which include the depression of lymphocytes (21). We found that not only total lymphocyte counts but also CD4^+^CD45RA^−^ T-cell counts were significantly lower in the second line and beyond patients. We speculated that the efficacy of ICIs in the second line and beyond would be affected by the previous treatments as and CD4^+^CD45RA^−^ T-cell counts. The results demonstrated that the correlation between CD4^+^CD45RA^−^ T cell and patients’ prognosis after ICIs was significantly higher in the second line and beyond (P = 0.006) than in the first line (P = 0.154). Therefore, the application of ICIs in the first-line therapy may help to better activate immune cells for anti-tumor efficacy.

Increased density of CD8^+^ TILs is an indicator of favorable prognosis for ICI treatment (22). Kamphorst et al. showed that patients with high levels of CD8^+^ T cell at baseline were strongly associated with OS benefit after receiving nivolumab (23). However, our study found no correlation between the absolute value of baseline CD8^+^ T cell and the prognosis of NSCLC. Notably, lower proportions of baseline CD8^+^ T/lymphocyte (%) were associated with better prognosis but did not affect the efficacy of ICIs. We hypothesized that patients responding well to treatment hold more CTL infiltrated into the tumor tissue. Therefore, less proportion of CD8^+^ T cell can be detected in peripheral blood. In addition to baseline expression status, early expansion of peripheral blood PD-1^+^CD8^+^ T cell may also correlate with anti-PD-1 inhibitors clinical efficacy (23). PD-1 inhibitors can interfere the PD-1 signaling and depress the upregulation of CBL-b ubiquitin ligase, thus boosting the proliferation of CD8^+^ T cell (24). PD-1 expressing peripheral blood CD8^+^ T cell, who possess highly similar TCR clones to TIL, had the ability to be activated by tumor cells and kill them (25, 26). We also explored the correlation between the changes of peripheral blood CD8^+^ T cell before and after ICI treatment. However, we did not find any correlation between the changes in CD8^+^ T cell and the prognosis. This may be explained by the fact that PD-1^+^ T cell represent only a small proportion of overall peripheral blood CD8^+^ T cell, whereas the proliferative burst of CD8^+^ T cell after PD-1 blockade was almost exclusively derived from the PD-1^+^CD8^+^ T-cell subset (27, 28).

The correlation between lymphocyte subsets and irAEs was also a research focus. Chaput et al. suggested that high levels of CD4^+^ T cell at baseline may predict a higher incidence of immune-associated colitis (29). Subudhi et al. found that high levels of CD8^+^ T cell may indicate an increased risk of irAEs (30). However, all of these findings suffered from small sample sizes and poor rigor. Our exploratory analysis results showed that patients who developed mild irAEs had higher levels of baseline CD4^+^ T cell and various CD4^+^ T cell subsets. In addition, the population of mild irAEs was highly overlapped with the population who obtained survival benefit. It validated the high correlation between the occurrence of irAEs and the efficacy of ICIs (31). In contrast to patients with mild irAEs, CD4^+^ T-cell levels in patients with severe irAEs, surprisingly, did not increase further but decreased to the level of non-irAE patients. The currently available mechanisms of irAEs include off-target of ICIs, cross-antigen reactions of T cells, and injury mediated by autoantibodies or cytokine (32). We ventured the hypothesis that severe irAEs and mild irAEs are mediated by two separate mechanisms, which needs to be explored and verified by further studies. Among all lymphocyte subsets we analyzed, only CD8^+^CD38^+^ T cell was positively correlated with the severity of irAEs. However, it did not show good predictive efficacy for severe irAEs.

This study was a retrospective research and inevitably existed with some deficiencies. First, the basic information of patients could not be well matched. For example, there was a significant correlation between the counts of CD4^+^CD45RA^−^ T cell and treatment lines. Although we tried to minimize them by performing subgroup analyses, they could not be completely eliminated. Second, the number of patients was insufficient, especially after performing the subgroup analyses. For instance, the immune monotherapy subgroup only contained 32 patients. Third, prolonging OS is the fundamental of oncology treatment. However, because of the lack of patients’ OS data, we evaluated the survival benefit only by ORR and PFS. In the future, a prospective study with large sample size is needed to further explore whether lymphocyte subsets can serve as the biomarker to predict the survival benefit and irAEs of ICI therapy.

Overall, NSCLC has entered the era of immunotherapy, but current biomarkers to predict patient response to ICIs are limited. Peripheral blood biomarkers provide a convenient, rapid, and non-invasive method for clinical diagnosis and disease prognosis. We propose that baseline peripheral blood CD4^+^CD45RA^−^ T-cell counts can be used as a biomarker for predicting the efficacy of ICIs, especially for the second line and beyond patients, whose tumor tissue acquisition is difficult. In addition, CD8^+^CD38^+^ T cell may be suggestive for the predicting the occurrence of irAEs. This study will pave the way for further prospective studies to use peripheral blood lymphocyte subsets as a non-invasive biomarker basis for ICI treatment in patients with NSCLC.

## Data Availability Statement

The original contributions presented in the study are included in the article/**Supplementary Material**. Further inquiries can be directed to the corresponding authors.

## Ethics Statement

The studies involving human participants were reviewed and approved by Ethics Review Committee of Peking Union Medical College Hospital. The patients/participants provided their written informed consent to participate in this study.

## Author Contributions

KM: Drafting the manuscript and revising it for important intellectual content. XZ, HW, XS, JN, WZ, JZ, YX, MC, and RP: Provision of clinical data, article revision, and correction. LZ and MZ: Substantial contributions to the conception or design of the work and final approval of the version to be published. All authors contributed to the article and approved the submitted version.

## Conflict of Interest

The authors declare that the research was conducted in the absence of any commercial or financial relationships that could be construed as a potential conflict of interest.

The reviewer J-LL declared a shared parent affiliation with the authors to the handling editor at the time of review.

## Publisher’s Note

All claims expressed in this article are solely those of the authors and do not necessarily represent those of their affiliated organizations, or those of the publisher, the editors and the reviewers. Any product that may be evaluated in this article, or claim that may be made by its manufacturer, is not guaranteed or endorsed by the publisher.

## Supplementary Material

Supplementary Figure 1 Absolute value of different lymphocyte subsets in benefit and non-benefit groups.

Supplementary Figure 2 Lymphocyte subsets changes after two cycles ICI therapy in benefit and non-benefit groups.

Supplementary Figure 3 Differences of CD4^+^CD45RA^−^ T cell between different treatment lines.

Supplementary Figure 4 Absolute value of different lymphocyte subsets in non-irAEs, mild-irAEs and severe-irAEs groups.

Supplementary Figure 5 ROC curve of CD8^+^CD38^+^ T cell for predicting severe irAEs.

## Abbreviations

NSCLC, non–small cell lung cancer; ICIs, immune checkpoint inhibitors; irAEs, immune-related adverse events; ORR, objective response rate; ECOG PS, eastern Cooperative Oncology Group performance status; PFS, progression-free survival; IQR, interquartile range; ROC, receiver operating characteristic curve; PD, progressive disease; AUC, area under the curve; TMB, tumor mutational burden; MSI, microsatellite instability; TIL, tumor-infiltrating lymphocytes; OS, overall survival.
