# Corrigendum: Peripheral blood lymphocyte subsets predict the efficacy of immune checkpoint inhibitors in non– small cell lung cancer

**DOI:** 10.3389/fimmu.2022.1111230

**Published:** 2022-12-08

**Authors:** Kang Miao, Xiaotong Zhang, Hanping Wang, Xiaoyan Si, Jun Ni, Wei Zhong, Jing Zhao, Yan Xu, Minjiang Chen, Ruili Pan, Mengzhao Wang, Li Zhang

**Affiliations:** Department of Pulmonary and Critical Care Medicine, Peking Union Medical College Hospital, Chinese Academy of Medical Sciences & Peking Union Medical College, Beijing, China

**Keywords:** immune checkpoint inhibitors (ICIs), lymphocyte subsets, CD4^+^CD45RA^−^ T cell, biomarker, CD8^+^CD38^+^ T cell

In the published article, there was an error in affiliation 1. Instead of “Department of Pulmonary and Critical Care Medicine, Peking Union Medical Hospital, Chinese Academy of Medical Science and Peking Union Medical College, Beijing, China”, it should be “Department of Pulmonary and Critical Care Medicine, Peking Union Medical College Hospital, Chinese Academy of Medical Sciences & Peking Union Medical College, Beijing, China”.

The authors apologize for this error and state that this does not change the scientific conclusions of the article in any way. The original article has been updated.

