# Supplementary figures and images for: Peripheral Blood Lymphocyte Subsets Predict the Efficacy of Immune Checkpoint Inhibitors in Non–Small Cell Lung Cancer
Source: Front Immunol. 2022 Jul 1;13:912180. doi: 10.3389/fimmu.2022.912180 (PMC9283649; doi:10.3389/fimmu.2022.912180)

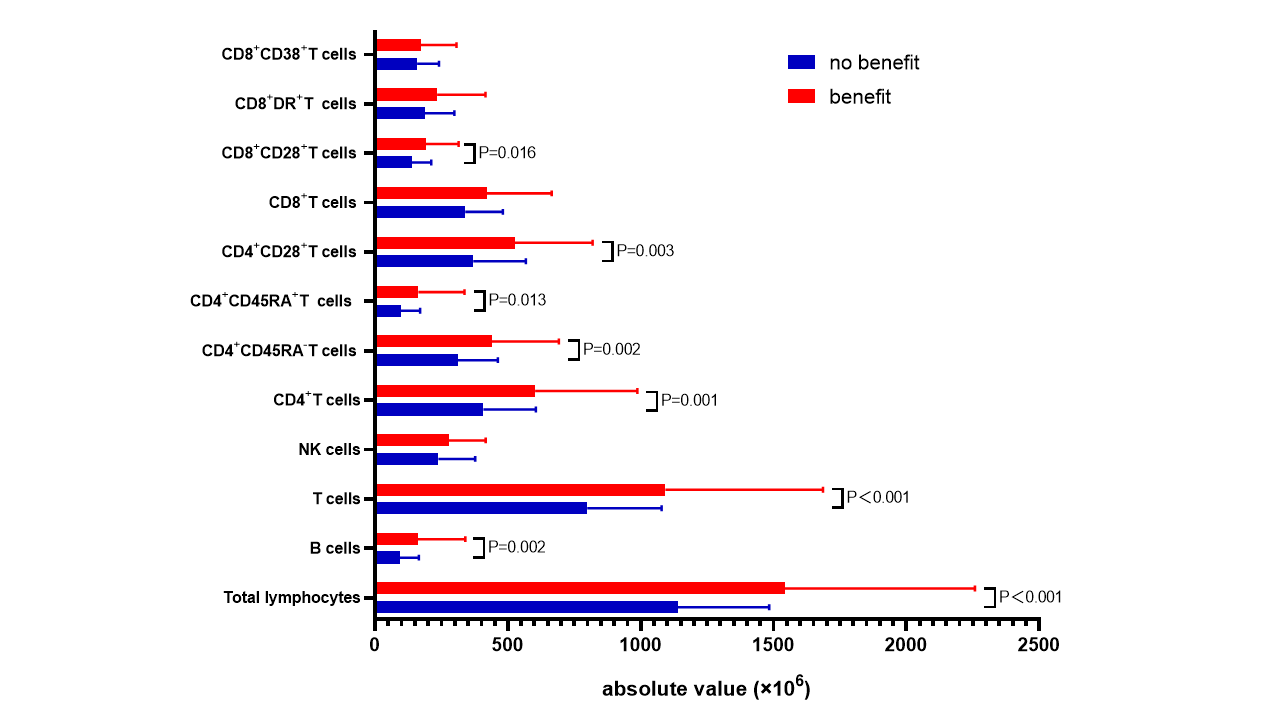

Supplement: Supplementary Figure 1 — Absolute value of different lymphocyte subsets in benefit and non-benefit groups. [file Image_1.tif]

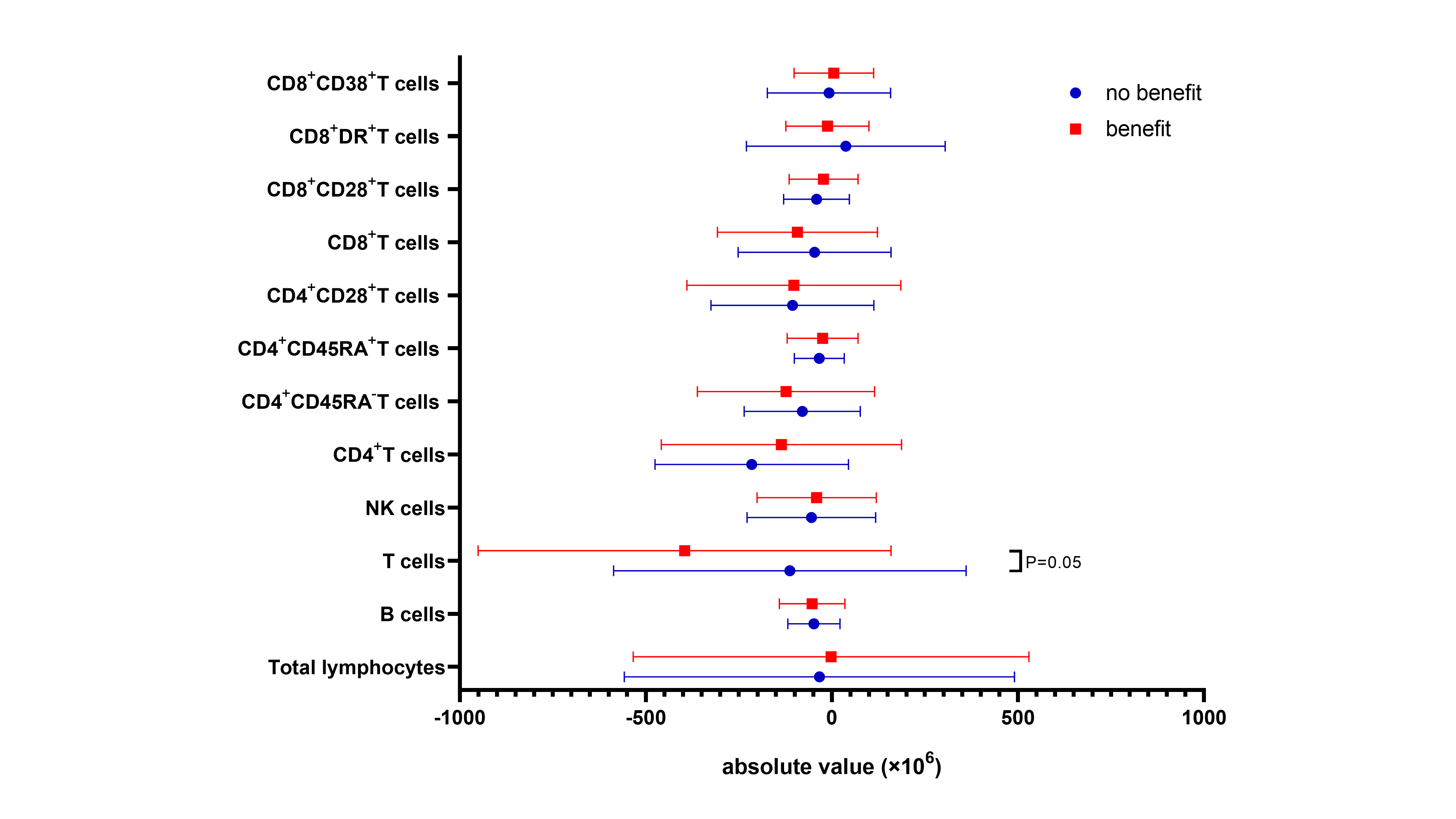

Supplement: Supplementary Figure 2 — Lymphocyte subsets changes after two cycles ICI therapy in benefit and non-benefit groups. [file Image_2.tif]

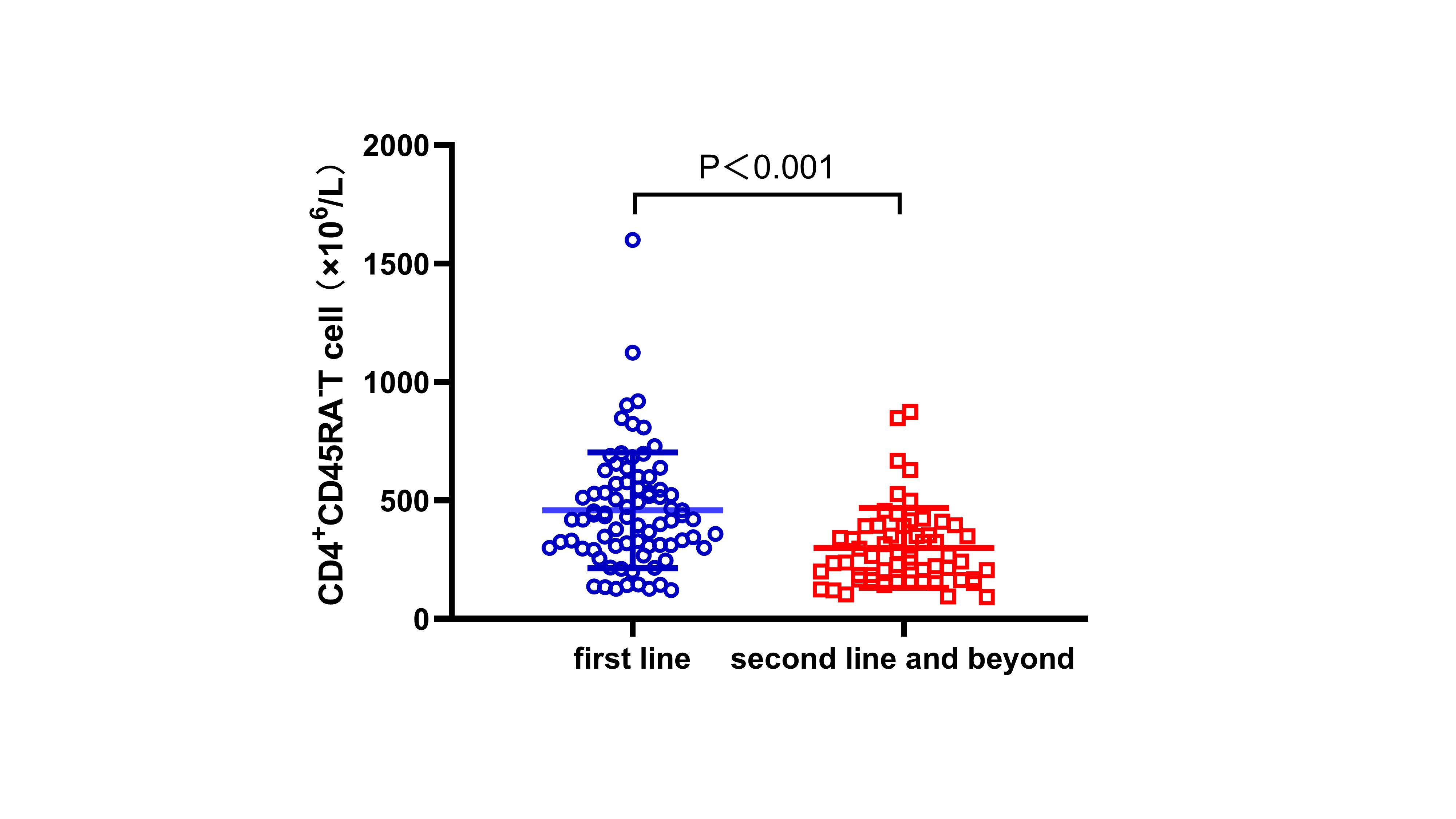

Supplement: Supplementary Figure 3 — Differences of CD4+CD45RA− T cell between different treatment lines. [file Image_3.tif]

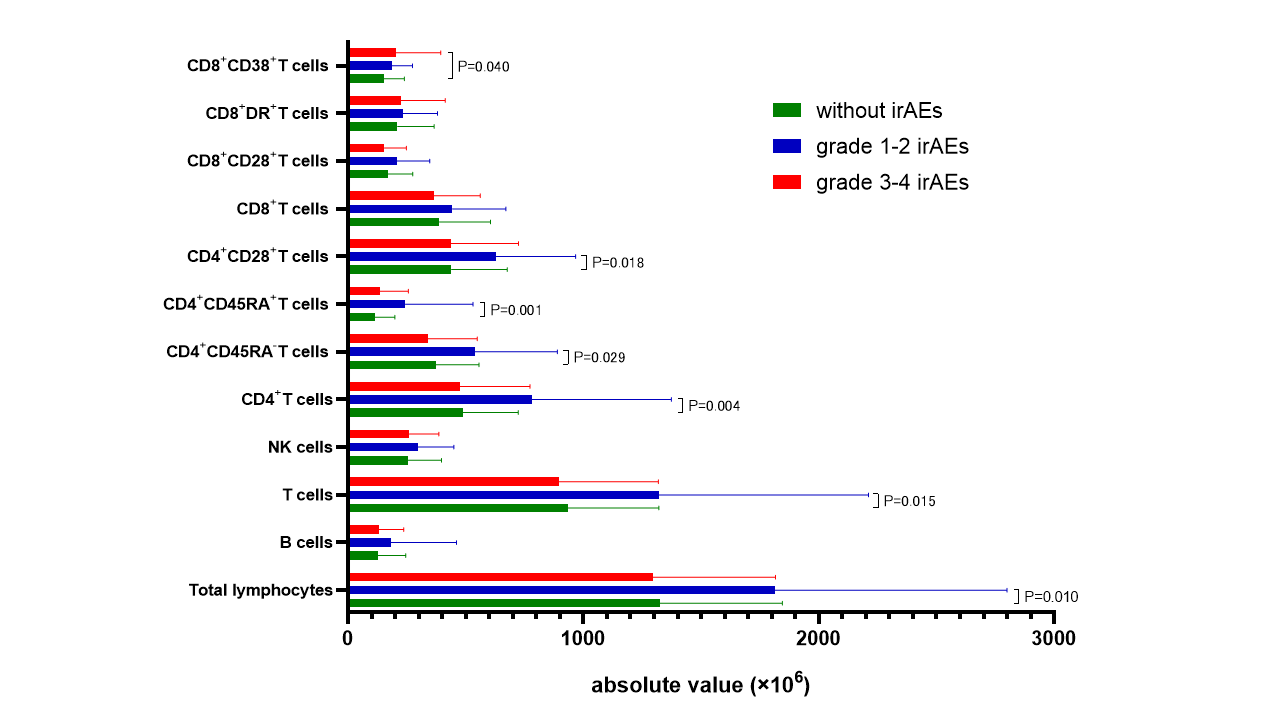

Supplement: Supplementary Figure 4 — Absolute value of different lymphocyte subsets in non-irAEs, mild-irAEs and severe-irAEs groups. [file Image_4.tif]

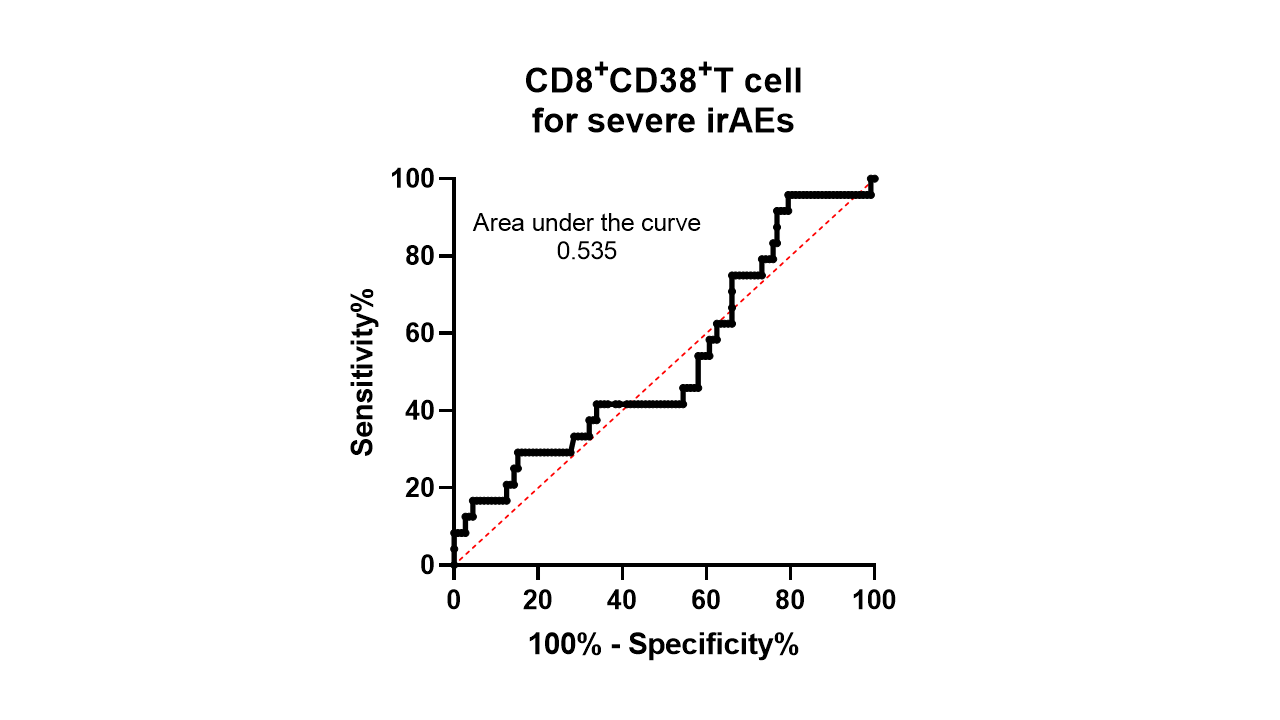

Supplement: Supplementary Figure 5 — ROC curve of CD8+CD38+ T cell for predicting severe irAEs. [file Image_5.tif]
